# Key mechanisms of affective disorders: CRC/TRR 393 project on mechanisms of emotion regulation, expectation, social cognition, and cognitive–behavioral rhythms

**DOI:** 10.1007/s00115-025-01920-9

**Published:** 2025-11-10

**Authors:** Philipp Kanske, Nina Alexander, Nadine Bernhardt, Stefan Ehrlich, Joachim Groß, Carsten Culmsee, Elisabeth J. Leehr, Andreas Jansen, Kay Jüngling, Philipp Ritter, Benjamin Straube, Ida Wessing, Tilo Kircher, Markus Wöhr

**Affiliations:** 1 Institute of Clinical Psychology and Psychotherapy, TUD Dresden University of Technology, Chemnitzer Str. 46, 01187 Dresden, Germany; 2 Department of Psychology, Babeș-Bolyai University, Cluj-Napoca, Romania; 3 Department for Psychiatry and Psychotherapy, Philipps-Universität Marburg, Marburg, Germany; 4 Department of Psychiatry and Psychotherapy, Technische Universität Dresden, Dresden, Germany; 5 Translational Developmental Neuroscience Section, Division of Psychological and Social Medicine and Developmental Neurosciences, Technische Universität Dresden, Dresden, Germany; 6 Institute for Biomagnetism and Biosignal Analysis, University of Münster, Münster, Germany; 7 Institute of Pharmacology and Clinical Pharmacy, Philipps-Universität Marburg, Marburg, Germany; 8 Center for Mind, Brain, and Behavior (CMBB), Philipps-Universität Marburg, Marburg, Germany; 9 Institute for Translational Psychiatry, University of Münster, Münster, Germany; 10 Institute of Physiology I, University of Münster, Münster, Germany; 11 Department of Child and Adolescent Psychiatry, University of Muenster, Muenster, Germany; 12 Experimental and Biological Psychology, Philipps-Universität Marburg, Marburg, Germany; 13 Research Unit Brain and Cognition, KU Leuven, Leuven, Belgium; 14 Leuven Brain Institute, KU Leuven, Leuven, Belgium

**Keywords:** Mood disorders, Depression, Bipolar disorder, Neuroimaging, Translational animal models, Stimmungsstörungen, Depression, Bipolare Störung, Neuronale Bildgebung, Translationale Tiermodelle

## Abstract

**Background:**

Although affective disorders are a major driver of disability worldwide, there is a lack of understanding of the mechanisms and modulating factors involved in the long-term disease trajectories.

**Objectives:**

Our goal is to determine key cognitive–emotional mechanisms in the domains of emotion regulation, expectation, social cognition, and cognitive–behavioral rhythms and their neurobiological correlates in the progression of affective disorders, including recurrences and remissions, chronicity, and functional decline.

**Materials and methods:**

In CRC/TRR 393, we will pursue a multi-level investigation of these four domains. Within the German Mental Health Cohort (GEMCO), these mechanisms and their influence on disease trajectories will be investigated longitudinally. Parallelized human and animal projects will enable an in-depth characterization of their neurobiological correlates.

**Results:**

By leveraging recent advancement in the modeling of complex, dynamic systems and machine learning techniques, we will be able to integrate human and animal data on the key cognitive–emotional mechanisms and their interplay with stressors and other modifying factors across disease trajectories.

**Conclusion:**

Gaining a deeper understanding of the cognitive–emotional mechanisms in the progression of affective disorders will help to predict symptom changes and course of illness as well as to identify key targets of intervention.

## Abstract (de)

**Hintergrund:**

Trotz der Bedeutung affektiver Störungen als Hauptursache für klinische Beeinträchtigungen weltweit besteht ein unzureichendes Verständnis der Mechanismen und modulierenden Faktoren, die langfristige Krankheitsverläufe bestimmen.

**Fragestellung:**

Ziel der vorliegenden Arbeit ist es, die zentralen kognitiv-emotionalen Mechanismen sowie ihre neurobiologischen Korrelate bei der Progression affektiver Störungen zu identifizieren. Dabei sollen Rückfälle, Remissionen, Chronifizierung und Funktionsverlust berücksichtigt werden.

**Material und Methoden:**

Im Rahmen des Collaborative Research Center/Transregio (CRC/TRR) 393 wird eine mehrstufige Untersuchung der 4 Domänen Emotionsregulation, Erwartungen, soziale Kognition und kognitiv-behaviorale Rhythmen durchgeführt. In der German Mental Health Cohort (GEMCO) werden diese Mechanismen und ihr Einfluss auf den Krankheitsverlauf longitudinal untersucht. Parallelisierte Human- und Tierprojekte ermöglichen eine detaillierte Charakterisierung ihrer neurobiologischen Korrelate.

**Ergebnisse:**

Durch die Nutzung aktueller Fortschritte in der Modellierung komplexer, dynamischer Systeme sowie maschinellen Lernens können human- und tierbezogene Daten zu den zentralen kognitiv-emotionalen Mechanismen und ihrer Interaktion mit Stressoren und anderen Modulatoren über den Krankheitsverlauf integriert werden.

**Schlussfolgerung:**

Das vertiefte Verständnis der kognitiv-emotionalen Mechanismen bei der Progression affektiver Störungen kann helfen, Verlauf und symptomatische Veränderungen besser vorherzusagen und gezielte interventionelle Ansätze zu identifizieren.

Affective disorders (AD) are highly prevalent mental disorders characterized by episodic and often chronic courses. Despite advances in understanding acute episodes and treatment responses, long-term disease trajectories remain poorly understood. The CRC/TRR 393 project addresses this gap by investigating dynamic, individual trajectories of AD, integrating human studies and rodent models. Key elements include real-time long-term monitoring, experimental studies focusing on cognitive–emotional mechanisms, and targeted interventions.

Cognitive–emotional mechanisms are central to theoretical models of AD, linking clinical presentation and neurobiology (Kircher et al., this issue, https://www.doi.org/10.1007/s00115-025-01886-8). Specifically, affective, cognitive, social, and somatic domains have been identified as key contributors to pathogenesis [12]; they are hypothesized to play a critical role in long-term disease trajectories (Ebner-Priemer et al., this issue) and as treatment targets (Leehr et al., this issue). From these domains, we delineated four key mechanisms: emotion regulation (ER), expectation (EXP), social cognition (SC), and cognitive–behavioral rhythms (CBR). Although largely studied in isolation, there is evidence of interactions among these mechanisms [20], underscoring the importance of integrated investigation of their role in symptom fluctuations, recurrences, remission, and overall disease course. Given their dynamic nature and association with brain function (Fig. 1), these mechanisms are promising candidates for identifying critical periods of symptom change. However, most existing research has focused on cross-sectional case–control comparisons, with relapse prediction models currently showing limited accuracy and external validity. In CRC/TRR 393, we aim to systematically examine these mechanisms during short-term symptom fluctuations over several years in naturalistic settings.

**Fig. 1:**
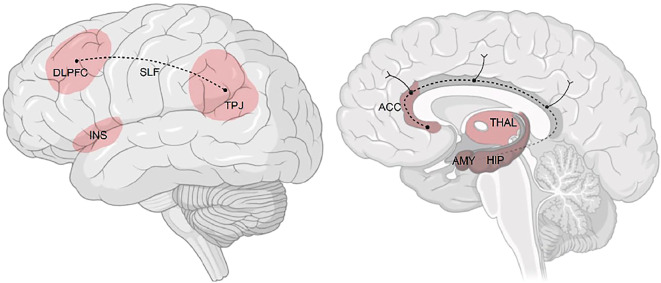
Schematic depiction of brain networks (regions in *red* and structural connections in black) associated with (1) affective disorders (AD) in cross-sectional studies, (2) disease trajectories in AD, and (3) cognitive–emotional mechanisms hypothesized to interact with periods of symptom change. *DLPFC* dorsolateral prefrontal cortex, *INS* insula, *TPJ* temporoparietal junction, *ACC* anterior cingulate cortex, *THAL* thalamus, *AMY* amygdala, *HIP* hippocampus, *SLF* superior longitudinal fasciculus. (With permission, ©T. Kircher, F. Stein)

## Emotion regulation (project B01)

Emotion regulation is broadly defined as the ability to manage emotions through automatic or controlled processes. Patients with AD exhibit less effective ER strategies [4, 8]. Contemporary models adopt a dynamic perspective, proposing that successful ER involves (1) variability in regulation strategies (e.g., size and diversity of the repertoire); (2) sensitivity to changing contextual demands; and (3) ER flexibility, i.e., the interplay between these factors [1, 2]. Neuroimaging studies have identified a frontolimbic network involved in both automatic and voluntary ER, comprising ventral (amygdala, subgenual anterior cingulate cortex [ACC]) and dorsal regions (dorsolateral prefrontal cortex, dorsal ACC, hippocampus; [6]). The role of this system in AD can be examined via functional magnetic resonance imaging (fMRI) in association with changes in the environment, e.g., stressful life events (SLEs) or during episodes of symptom change.

**Fig. 2:**
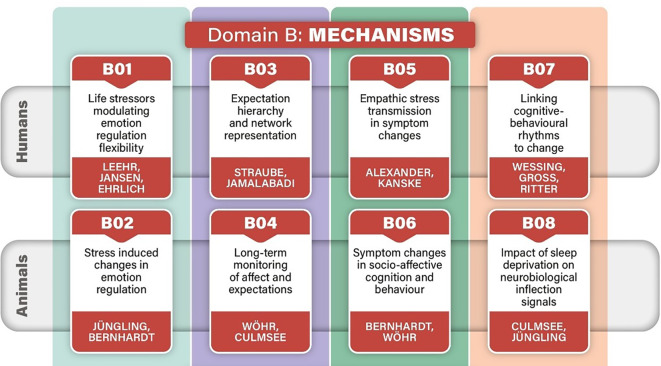
Overview of B projects in the CRC/TRR 393 project conducted with humans (GEMCO and different subsamples) and animals. A core assessment covering parts of the mechanisms is also completed in the full cohort (see Kircher et al., in this issue, 10.1007/s00115-025-01886-8)

Emotion regulation will be assessed in the full German Mental Health Cohort (GEMCO) and in depth in a subsample (see Fig. 2 for an overview of B projects) using a baseline fMRI measurement including an emotion perception as well as an ER choice task, questionnaires, comprising well-established ER questionnaires as well as newer ones, and a 2-week intensive ecological momentary assessment (EMA), evaluating emotional events and regulatory activities and their success. Over the following 18 months, the occurrence of SLEs and subsequent changes in ER will be monitored by reinviting patients to the laboratory in the event of an SLE (Ebner-Priemer et al., this issue). The goal is to investigate the relevance of emotion perception and ER flexibility for disease trajectories and to explore the predictive capacity of adaptive emotional functioning following SLEs for clinical outcomes. It is expected that a wide ER strategy repertoire with an adaptive and thus flexible ER strategy choice (assessed via EMA and during the fMRI paradigm) will serve as a resilience factor after SLEs and thus be associated with less deterioration in affective symptoms. Within the fMRI paradigm, higher emotional reactivity to negative stimuli (as indexed, for instance, by more negative ratings, increased fMRI limbic activity, and altered pupil dilation) will be associated with more severe changes of affective symptoms after an SLE. Furthermore, a more frequent choice of distraction versus reappraisal during the ER choice paradigm, along with lower effectiveness of ER measured via functional frontolimbic connectivity and emotion ratings, is assumed to be associated with more severe changes of affective symptoms after an SLE.

## Expectation (project B03)

Expectations are explicit or implicit processes that are directed toward future events or experiences, focusing on their occurrence or absence. In AD, maladaptive EXPs have been widely associated with symptom manifestation [15]. We and others have developed various paradigms in which individuals learn and modify EXPs about upcoming aversive events across different timescales. Notably, maladaptive EXPs are believed to be strong predictors of symptom fluctuations in AD. For example, patients with major depressive disorder (MDD) frequently display negative future expectancies and difficulties in adjusting reward EXPs, which contribute to reduced motivation and maintenance of depressive symptoms. Such maladaptive EXPs are thus not only correlates but strong predictors of symptom fluctuation and recurrence. Neuroimaging studies have linked EXPs to prefrontal regions, particularly the dorsolateral prefrontal cortex and ACC, as well as subcortical limbic structures.

Expectations will be assessed in the full GEMCO and in depth in subsamples with MDD. Furthermore, fMRI and longitudinal assessments will examine how negative EXPs influence neural circuits involved in fear conditioning, social decision-making, and future-directed thinking. The EMA assessments will allow us to track changes in EXPs over time and assess how they predict clinical trajectories and inflection signals. We will also evaluate the effectiveness of a targeted EXP-focused intervention in modifying both general and situation-specific EXPs and their neural correlates [10]. Experimental paradigms will distinguish between experience-based and instruction-based EXPs. For example, an interactive game with multiple playing partners will be used to investigate how instructions about playing partners and experiences with the playing partner shape social decisions and EXPs about future interactions. Usually, EXP violations (e.g., due to new experiences or instructions) lead to EXP change. However, in patients with MDD, often more rigid negative EXPs persist even in the context of positive experiences or instructions (cognitive immunization). Our comprehensive approach aims to elucidate the mechanistic role of EXPs in MDD progression and identify predictive markers of symptom change and recovery.

## Social cognition (project B05)

Social interactions are highly influential for both mental and physical health in general and particularly in AD [18]. Recent evidence indicates that social stress transmission—physiological stress responses caused by observing others’ distress—may be a key factor [5]. This suggests that witnessing stressful events within one’s social network can contribute to symptom fluctuations and recurrence in AD. Social cognition mechanisms underlying social stress transmission involve affective and cognitive pathways: Affect-sharing relies on regions within the salience network, including the anterior insula and ACC, while perspective-taking engages areas of the theory-of-mind network, particularly the temporoparietal junction [19]. Several studies indicate that sociocognitive functions are disrupted in AD, but the precise role of these functions in social stress transmission, symptom change, and relapse remains to be fully elucidated.

In CRC/TRR 393, we will investigate empathic stress transmission across biological pathways—endocrine, cardiovascular, immunological, neural—in both experimental (Empathic Trier Social Stress Test) and naturalistic settings. Using stress tests, (f)MRI, self-reports, genotyping, and e‑diary assessments, we will examine the role of stress transmission in depressive symptom changes in GEMCO and in a subsample. We will focus particularly on dyads of MDD patients and their partners to explore how emotional and social closeness influences stress transmission. Our goal is to identify social interaction patterns that contribute to the relapse and onset of MDD, informing personalized risk assessments and interventions.

## Cognitive–behavioral rhythms (project B07)

Cognitive–emotional functioning and neuroplasticity are strongly influenced by circadian rhythms, especially sleep [16]. Dysregulation of these rhythms, particularly REM sleep, is considered a key pathogenic mechanism contributing to the onset and modulation of symptom change in AD. Alterations in circadian timing are among the most frequent early indicators of impending symptom exacerbation prior to an episode [17]. During sleep and wakefulness, electrophysiological recordings reveal rhythms that are notably disrupted in AD. Moreover, various endocrine, inflammatory, and cognitive disturbances observed in patients correspond to abnormalities seen in experimental models involving shifts in circadian timing.

In CRC/TRR 393, we will investigate the link between sleep–activity patterns and the recurrence of affective episodes. We will initially monitor self-reported sleep, smartphone activity, wake EEG, and mood in GEMCO across a 2-year period. A subgroup will undergo more intensive, objective monitoring, including continuous actigraphy for 24 months, alongside sleep EEG and wake MEG/EEG measurements at the beginning and after a significant mood change. Our protocol combines baseline and post-inflection sleep EEG, wake EEG, MEG, and actigraphy to test the hypothesis that thalamocortical oscillatory dynamics—indexed by REM measures and spindle density—serve as state- and trait-like predictors of relapse vulnerability and subthreshold affective symptoms. By analyzing the interplay between sleep–activity rhythms, brain oscillations, cognitive–emotional functions, and mood symptoms over time, we aim to uncover the mechanisms that confer vulnerability to, and drive the recurrence of, affective episodes.

## Animal studies (projects B02, B04, B06, B08)

The validity of rodent models for mental disorders strongly relies on their behavioral phenotype, making optimized behavioral phenotyping crucial for advancing translational research (Kircher et al., in this issue, https://www.doi.org/10.1007/s00115-025-01886-8). In particular, models that enable the detection of inflection signals and long-term trajectories with high temporal resolution for studying key cognitive–emotional mechanisms associated with AD are warranted.

The four animal projects in CRC/TRR 393 apply a gene × environment (GxE) approach by exposing *Cacna1c *haploinsufficient mice and rats to relevant environmental risk factors, e.g., sleep deprivation, restraint stress, or social defeat, as compared to non-stressed controls. Single nucleotide polymorphisms in *CACNA1C, *a top-ranked cross-disorder risk gene, are linked to multiple mental disorders, including AD [9]. *CACNA1C *encodes the α1C subunit of the L‑type calcium channel (LTCC) Cav1.2, the dominant LTCC in the brain, yet the consequences of *CACNA1C* single nucleotide polymorphisms are still not fully understood. Wild-type littermates will serve as controls.

**Fig. 3:**
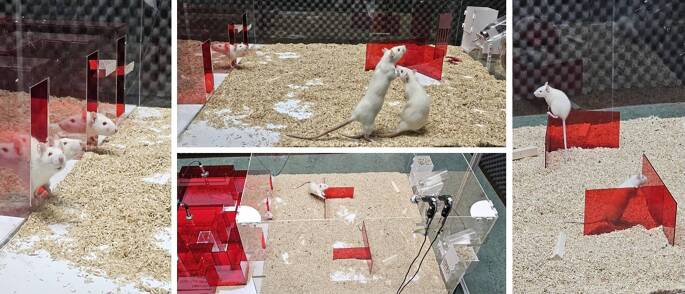
Semi-natural environment for detecting inflection signals in rats. (With permission, © Fábio J. Sousa)

Importantly, our GxE approach applied in *Cacna1c *haploinsufficient mice and rats includes novel experimental strategies to identify inflection signals. It is characterized by two key features. First, we assess AD-relevant behavioral phenotypes in rodents housed in semi-natural environments (Fig. 3), which promote natural behaviors and enhance detection of AD-relevant phenotypes. Second, we perform continuous and intermittent long-term monitoring of relevant behavior and assess concomitant changes in the four cognitive–emotional mechanisms, with individual components of the rodent behavioral profile offering insight into multiple if not all four cognitive–emotional mechanisms.

This includes socio-affective communication through ultrasonic vocalizations (USVs; [3]), a key element of the rich natural behavior repertoire of rodents and thus our experimental strategy. For example, in rats, two main USV types are particularly important for assessing AD-relevant trajectories: Aversive 22-kHz USVs occur during and in anticipation of stressful events, e.g., predator exposure; appetitive 50-kHz USVs occur during and in anticipation of rewarding events, e.g., social play, or in response to psychostimulants [22]. As social signals (SC) reflecting changes in the affective state (ER) in anticipation of expected outcomes (EXP) and characterized by a prominent circadian rhythm (CBR), USVs represent a unique and key component of the rodent behavioral profile. This component can serve as “readout” for all four cognitive–emotional mechanisms.

Quantitative assessment of USVs provides a robust measure of socio-affective functioning in the sender, ideal for modeling human mental disorders characterized by alterations in ER and EXP. While 22-kHz USVs reflect negative affect akin to anxiety and fear, 50-kHz USVs dubbed “rat laughter” [14] reflect positive affect similar to joy and happiness. Their emission is a central element of the anticipatory emotional response. Particularly relevant for modeling BD is the observation that 50-kHz USVs can serve as a marker of mania-like elevated mood and hypersociability. For example, amphetamine and sleep deprivation enhance 50-kHz USVs, which can be blocked by lithium, the gold standard for treating BD [22]. In CRC/TRR 393, 50-kHz USVs therefore serve as a graded measure for positive affect. While a lack of 50-kHz USVs can be interpreted as a sign of anhedonia relevant to MDD, high 50-kHz USV emission can be seen as a marker of mania-like elevated mood relevant to BD.

Quantitative assessment of behavioral responses evoked by USVs provides a reliable indicator of socio-affective functioning in the receiver, ideal for modeling human mental disorders characterized by alterations in SC. In fact, 22-kHz and 50-kHz USVs serve as socio-affective signals with distinct communicative functions [3]. In conspecific receivers, 22-kHz USVs trigger a fear-like response and amygdala activation in line with an alarming function, while 50-kHz USVs evoke social approach and dopamine release in the nucleus accumbens in line with a social contact function [21]. In CRC/TRR 393, ultrasound playback is combined with electrophysiological recordings [7] to identify brain signatures associated with changes in SC and ultimately inflection signals.

Importantly, genetic *Cacna1c *rodent models display behavioral phenotypes with relevance for MDD [23]. Specifically, *Cacna1c *haploinsufficient rats display robust deficits in socio-affective communication, e.g., deficits in 50-kHz USV emission during social play and reduced social approach elicited by 50-kHz USV playback, together with alterations in cognitive functioning and anhedonia. Behavioral phenotypes are modulated by environmental factors, including juvenile social isolation, known to induce MDD-like cognitive impairment and social withdrawal. This is paralleled by changes in the brain microRNA profile, mediating environmental impact. Specifically, MDD-like phenotypes were associated with an upregulation of miR-134, a negative regulator of synaptic plasticity, while a lack of a microRNA cluster including miR-134 resulted in an antidepressant-like phenotype [11].

In addition to behavioral assessment and concomitant electrophysiological recordings, changes in immune-metabolic parameters, including altered mitochondrial parameters and cytokine signatures, will be studied. This strategy is based on our observation that behavioral phenotypes displayed by *Cacna1c *haploinsufficient rats are associated with changes in mitochondrial resilience to oxidative stress [13]. It is hypothesized that neurobiological features of AD may change in relation to inflection signals and long-term trajectories. Therefore, we will investigate neurobiological alterations associated with inflection signals in mice and rats, focusing on changes in metabolic signatures attributed to altered mitochondrial function in peripheral immune cells, brain microglia, and neurons.

## Conclusion and outlook

These integrative, multi-level projects advance our understanding of the cognitive–emotional mechanisms underlying AD trajectories. By combining longitudinal human cohort data with animal models and cutting-edge modeling approaches (Dannlowski et al., this issue), we will elucidate the interactions between ER, EXP, SC, and CBR. Recent developments in complex systems modeling and machine learning will enable the integration of findings across species and scales, offering novel insights into the neural and neurobiological underpinnings. These mechanistic insights hold considerable potential for improving predictive models of symptom change, identifying critical intervention targets, and developing personalized treatment strategies. Ultimately, this research paves the way toward more effective prevention and early intervention in AD, translating mechanistic understanding into tangible benefits for patients.

## Practical conclusion

- Future management of affective disorders (AD) may benefit from a focus on key cognitive–emotional mechanisms such as emotion regulation (ER), expectation (EXP), social cognition (SC), and circadian cognitive–behavioral rhythms (CBR).
- Longitudinal monitoring of these mechanisms could enhance the prediction of symptom trajectories and support early intervention strategies.
- Interventions aimed at increasing ER flexibility, addressing dysfunctional EXP, and integrating SC training and chronotherapeutic approaches (e.g., sleep regulation) could contribute to stabilizing or even improving long-term illness courses.
- Incorporating neurobiological markers (e.g., neuroimaging, mitochondrial function) into assessments may support more personalized treatment approaches in the future.

## Declarations

All national guidelines on the keeping and handling of laboratory animals were complied with, and the necessary approvals from the relevant ethics committee have been obtained.

The supplement containing this article is not sponsored by industry.
